# Computational analysis of protein-protein interfaces involving an alpha helix: insights for terphenyl–like molecules binding

**DOI:** 10.1186/2050-6511-14-31

**Published:** 2013-06-14

**Authors:** Adriana Isvoran, Dana Craciun, Virginie Martiny, Olivier Sperandio, Maria A Miteva

**Affiliations:** 1 Department of Biology and Chemistry, West University of Timisoara, 16 Pestalozzi, Timisoara 300115, Romania; 2 Advanced Environmental Researches Laboratory, 4 Oituz, Timisoara 300086, Romania; 3 Teacher Training Department, West University of Timisoara, 4 Blvd. V. Parvan, Timisoara 300223, Romania; 4 Université Paris Diderot, Sorbonne Paris Cité, Molécules Thérapeutiques in silico, Inserm UMR-S 973, 35 rue Helene Brion, Paris 75013, France; 5 INSERM, U973, Paris F-75205, France

## Abstract

**Background:**

Protein-Protein Interactions (PPIs) are key for many cellular processes. The characterization of PPI interfaces and the prediction of putative ligand binding sites and hot spot residues are essential to design efficient small-molecule modulators of PPI. Terphenyl and its derivatives are small organic molecules known to mimic one face of protein-binding alpha-helical peptides. In this work we focus on several PPIs mediated by alpha-helical peptides.

**Method:**

We performed computational sequence- and structure-based analyses in order to evaluate several key physicochemical and surface properties of proteins known to interact with alpha-helical peptides and/or terphenyl and its derivatives.

**Results:**

Sequence-based analysis revealed low sequence identity between some of the analyzed proteins binding alpha-helical peptides. Structure-based analysis was performed to calculate the volume, the fractal dimension roughness and the hydrophobicity of the binding regions. Besides the overall hydrophobic character of the binding pockets, some specificities were detected. We showed that the hydrophobicity is not uniformly distributed in different alpha-helix binding pockets that can help to identify key hydrophobic hot spots.

**Conclusions:**

The presence of hydrophobic cavities at the protein surface with a more complex shape than the entire protein surface seems to be an important property related to the ability of proteins to bind alpha-helical peptides and low molecular weight mimetics. Characterization of similarities and specificities of PPI binding sites can be helpful for further development of small molecules targeting alpha-helix binding proteins.

## Background

Protein-Protein Interactions (PPIs) are key to many cellular processes. Abnormal PPIs contribute to many disease states and as such, PPIs represent today a new class of drug targets essentially unexploited for drug discovery. Indeed, the size of the human interactome has been estimated to be between 300,000 [1] and 650,000 interactions [2]. In the last decade many studies have been performed in order to target PPIs [3]. Several small-molecule inhibitors of PPIs have been demonstrated therapeutic potential [4-8]. However, efficient targeting of PPIs is still being considered as an important challenge [3,9,10]. In contrast to enzyme-substrate interactions, protein-protein recognition often occurs through flat surfaces or wide shallow grooves. Recent structural analyses of PPI interfaces and small molecules disrupting PPIs suggested that such ligands might mimic the structural characteristics of the protein partner [6,11]. To facilitate the discovery of new PPI small-molecule inhibitors, the characterization of PPI interfaces [12,13] and the prediction of putative ligand binding sites are essential. Physicochemical properties of both ligand and protein are key to mediate the binding [14], such as cavity sizes, shape complementarity, electrostatic potential and hydrophobicity [12,15].

The role of alpha-helical peptides in mediating many PPIs is well demonstrated and development of small organic molecules mimicking such peptides becomes important [16]. Recent studies have been carried out on the whole Protein Data Bank (PDB) in order to establish a druggability profile of alpha-helix mediated PPIs and to predict which of them could bind a small molecule [17]. More specifically, terphenyl and its derivates are small organic molecules [18-26] mimicking one face of an alpha-helical peptide, *i.e.* the side chains of three key residues occupying positions *i, i+3* and *i+7*[25,26] or *i, i+4* and *i+7*[20] of the bound helix. It has been suggested that terphenyl compounds can serve as pharmacological probes because they are membrane permeable [22]. Terphenyl 1 and 2, which mimic the calmodulin binding face of smooth muscle myosin light chain kinase (smMLCK), have been shown to inhibit the interactions of calmodulin (CaM) with the enzyme 3'-5'-cyclic nucleotide phosphodiesterase (PDE) and with the helical peptide C20W of the plasma membrane calcium pumps [18]. Following the similarity between the calmodulin and human centrin 2 (HsCen2) alpha-helix binding sites, we recently suggested that terphenyl 2 might also inhibit the interaction between HsCen2 and a 17 residues peptide of *Xeroderma Pigmentosum Group C* (XPC) protein [27]. Terphenyl derivates mimicking the alpha-helical structure of p53 N-terminal peptide inhibit the p53-MDM2 [22] and the p53-HDM2 interactions [21]. These molecules also mimic the alpha-helical region of Bak BH3 domain, which binds BCL-X_2,_ thus disrupting the BCL-X_2_/Bak interaction [19,20,24].

In this work we performed a computational analysis in order to evaluate several key physicochemical and surface properties of proteins known to interact with alpha-helical peptides or to bind terphenyl and its derivatives. We calculated the binding pocket volumes and the fractal dimensions of the surface cavities for the entire protein and for the binding pockets. We identified several similarities and specificities characterizing such protein binding sites that can be helpful for future development of more efficient small-molecule inhibitors targeting alpha-helix binding proteins.

## Methods

In this study we compared the sequence and surface properties of the investigated proteins. In order to analyze the sequence similarities we performed sequence alignment using the CLUSTALW software [28]. Interacting residues at the protein-protein interface in terms of contact distances were found using the ContPro online freely available tool [29]. We identified the protein residues interacting with the three key residues of the alpha-helical peptide (occupying positions *i, i+3* and *i+7* or *i, i+4* and *i+7*) those relative positions are mimicked by terphenyl and its derivatives. The distance threshold was set to 5 Å for the side chain atoms.

In order to evaluate the protein surface properties, the bound peptide was removed for each complex. The surface characteristics of the entire protein and those of the peptide-binding cavity were analyzed. Using the approach of the fractal geometry we quantitatively described the surface roughness for the entire protein and for the binding cavity, expressed by global surface fractal dimension (D_S_) and local surface fractal dimension (D_L_), respectively. In order to calculate the surface fractal dimension we used the method proposed by Lewis and Rees [30] based on the scaling law between the surface area (SA) and the radius of the rolling probe molecule (R) on the surface, i.e. SA is proportional to the radius to the power 2-Ds:

(1) $$\mathrm{SA}\sim R^{2-D_{S}}$$

The surface fractal dimension was determined from the slope of the double logarithmical plot of SA versus R. The surface area of the protein was computed using the on-line available software GETAREA [31]. Probe radii of 1, 1.2, 1.4, 1.6, 1.8 and 2 Å were used. For the proteins cavities, the same algorithm was employed using the CASTp software [32]. Hydrophobicity and local hydrophobic density for binding pockets were determined using Fpocket [33]. Pocket volumes were computed using CASTp [32].

Molecular docking of terphenyl 2 was performed into the alpha-helical binding sites of calmodulin (code entry 2O5G) and troponin C (code entry 1A2X) using AutoDock 4.2 [34]. The input files preparation and docking analysis were carried out using AutoDockTools. Grid maps were centered in the alpha-helix binding site for both structures. Grids sizes were 126 Å x 126 Å x 126 Å with a grid spacing of 0.33 Å for calmodulin and 126 Å x 126 Å x 126 Å with a grid spacing of 0.28 Å for troponin C. Ligand conformational searching was performed using Lamarckian genetic algorithm and all ligand torsion angles were flexible. The following docking parameters were used: 250 Lamarckian genetic algorithm runs, a population size of 250, a maximum of 2 500 000 energy evaluations and a maximum of 27000 generations.

Figures were prepared using PyMol [35] and CHIMERA software [36].

## Results and discussions

### Sequence-based analysis

We analyze several proteins interacting with alpha-helical peptides, some of them being known to bind also terphenyl and/or its derivatives. To characterize and compare their surface properties we examine the sequences and the three dimensional (3D) structures of the complexes formed by the protein and the bound peptide. The 3D structures are retrieved from the PDB [37], the entry codes being presented in Table 1. Most of the structures are crystallographic. Two NMR structures are also used: the C-terminal domain of human centrin 2 in complex with the repeat sequence of human Sfi 1 and the human BCL-XL in complex with the BAK peptide.

**Table 1:** Protein – alpha-helical peptide complexes

**Protein complex**	**PDB code Resolution**	**SwissProt code**	**Interacting residues of the bound alpha-helix**
Chicken calmodulin in complex with smooth muscle myosin light chain kinase (smMLCK)	2O5G^*^	P62149	TRP5, THR8, VAL12
	1.08 Å		
Human calmodulin in complex with a mutant peptide of human DRP-1 kinase	1ZUZ	P62158	TRP305, PHE309, VAL312
	1.91 Å		
Human calmodulin in complex with CAV1.1 IQ peptide	2VAY^*^	P62158	THR526, ILE529, PHE533
	1.94 Å		
Human calmodulin in complex with CAV2.2 IQ peptide	3DVE	P62158	MET854, VAL857, MET161
	2.35 Å		
E Coli calmodulin in complex with RS20 peptide of smMLCK	1QTX	-	TRP5, THR8, VAL12
	1.65 Å		
Rat calmodulin in complex with NMDA receptor NR1C1peptide	2HQW	P62161	PHE880, THR884, LEU887
	1.90 Å		
Human centrin 2 in complex with the centrin binding region of XPC protein	2GGM	P41208	TRP848, LEU851, LEU855
	2.35 Å		
C-terminal domain of human centrin 2 in complex with a repeat sequence of human Sfi 1	2K2I	P41208	LEU651, LEU655, TRP658
	NMR		
Scherffelia dubia centrin in complex with smMLCK peptide	3KF9	Q06827	TRP4, PHE8, VAL11
	2.60 Å		
Human BCL-XL in complex with BAK peptide	1BXL^*^	Q07817	VAL574, LEU578, ILE581
	NMR		
Human E3 ubiquitin-protein ligase MDM2 in complex with p53 tumor transactivation domain (fragment 17-125)	1YCR^*^	Q00987	PHE19, TRP23, LEU26
	2.60 Å		
Rabbit cardiac troponin C in complex with a fragment (residues 1-47) of cardiac troponin I	1A2X	P02586	LEU17, MET21, ILE24
	2.30 Å		

*known to be disrupted by terphenyl or its derivatives.

**Figure 1:**
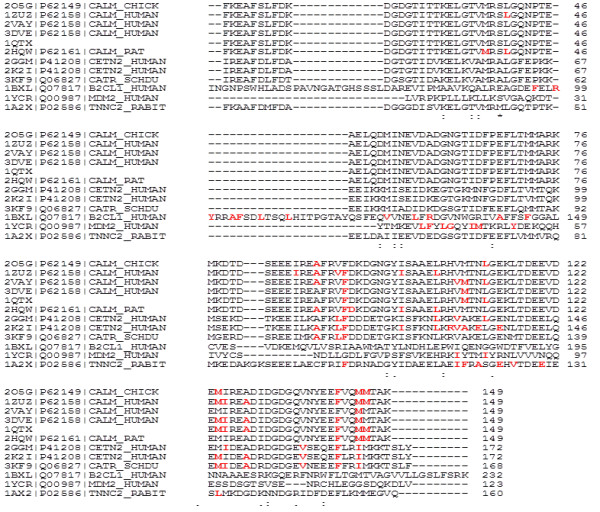
**Sequence alignment of alpha-helix binding proteins.** The amino acid residues interacting with alpha-helical peptides are presented in red.

Multiple sequences alignment (Figure 1) shows low sequence identity for the most of the analyzed proteins (shown in Table 2) both for the entire sequences and for the binding areas. The binding areas included all residues of the protein interacting with the alpha-helical peptide. Chicken, human, E. coli and rat calmodulin have very similar sequences (rat, chicken and human calmodulin are 100% identical; E coli has 98% identity with the others). For BCL-XL and human ubiquitin carboxyl-terminal hydrolase MDM2 only those fragments of sequences that are present in the 3D structures are considered. There is a high similarity only between the calmodulin, centrin 2 and troponin C sequences.

**Table 2:** Sequence identity (in %) between the considered proteins (the binding area/entire protein)

**Protein/ sequence identity**	**Human calmodulin**	**Human centrin 2**	**Scherffelia dubia centrin**	**Human BCL-X**_**2**_	**Human E3 ubiquitin-protein ligase MDM2**
Human centrin 2	54/50				
Scherffelia dubia centrin	56/55	90/74			
Human BCL-X_2_	5/7	5/5	5/8		
Human E3 ubiquitin-protein ligase MDM2	5/4	5/10	7/6	9/5	
Rabbit cardiac troponin C	57/51	57/34	37/32	5/9	5/19

The binding area was defined here as all residues of the protein interacting with the helical peptide.

### Structure-based analysis

**Figure 2:**
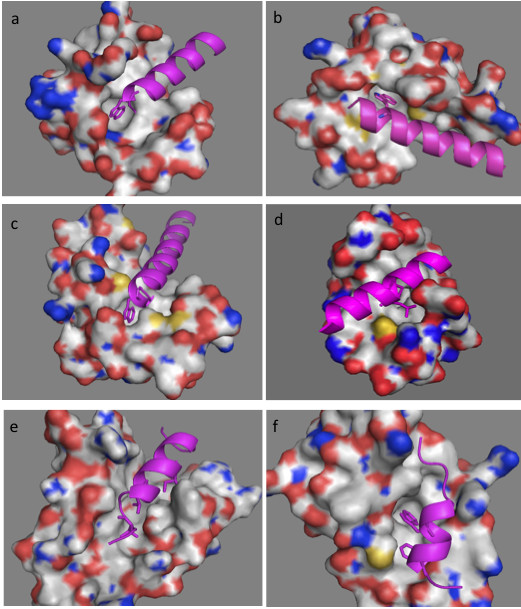
**3D structures of the complexes formed by:** (**a**) human centrin 2 and a 10 residue peptide of Xeroderma Pigmentosum group C protein, code entry 2GGM. (**b**) chicken calmodulin and smooth muscle myosin light chain kinase (smMLCK), code entry 2O5G. (**c**) scherffelia dubia centrin and smMLCK peptide, code entry 3KF9. (**d**) rabbit cardiac troponin C and a fragment of cardiac troponin I, code entry 1A2X. (**e**) human BCL-XL and BAK peptide, code entry 1BXL. (**f**) human E3 ubiquitin-protein ligase MDM2 and p53 tumor transactivation domain, code entry 1YCR. All proteins are shown as surface in atom color type (C and H-white, N – blue, O -red, S – yellow) and ligands are shown in magenta cartoon with hydrophobic interacting residues given as sticks.

**Figure 3:**
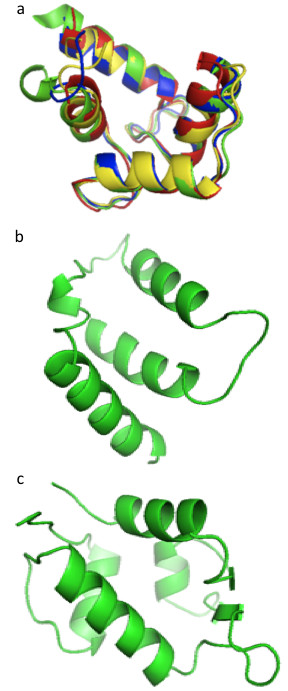
**3D structures of alpha-helix binding domains.** (**a**) Superposition of the alpha-helix binding regions of chicken calmodulin (red, code entry 2O5G), HsCen2 (blue, code entry 2GGM), scherffelia dubbia centrin (green, code entry 3KF9) and rabbit troponin C (yellow, code entry 1A2X). (**b**) Structure of human BCL-XL binding domain (code entry 1BXL). (**c**) Structure of human E3 ubiquitin-protein ligase MDM2 (code entry 1YCR) binding domain.

Figure 2 illustrates the complexes’ structures of six alpha-helix binding proteins. In all shown complexes, bulky hydrophobic residues of the bound peptide anchor into the protein binding pocket. Following the sequence similarities we superimposed the alpha-helix binding regions structures of calmodulin, human centrin 2, scherffelia dubia centrin and rabbit troponin C (Figure 3a). Strong structural homology for binding regions is seen following the sequence similarity of these proteins. Figure 3b and 3c illustrate the binding pockets of BCL-XL and human E3 ubiquitin-protein ligase MDM2, respectively.

**Figure 4:**
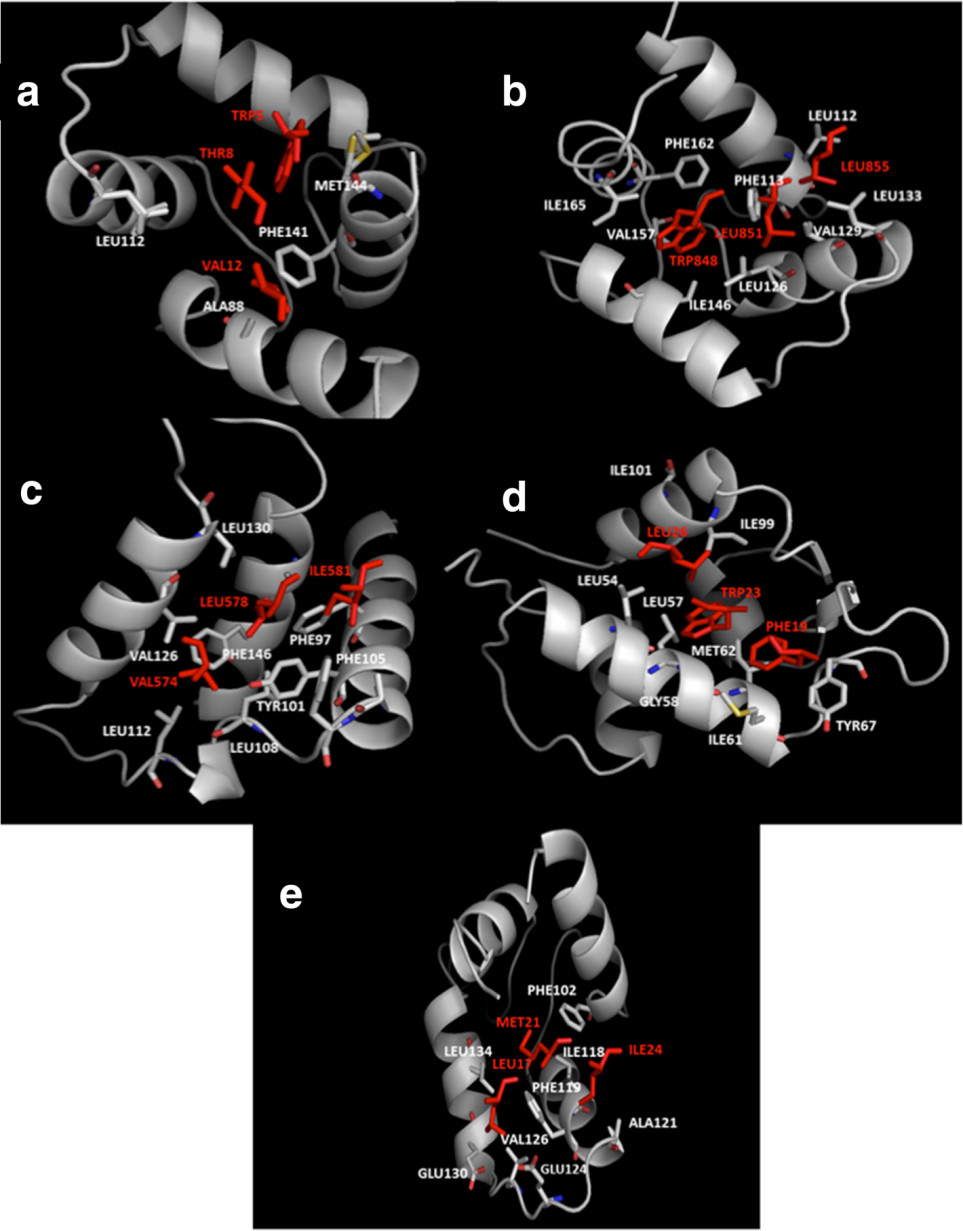
**Illustration of the interacting residues (in sticks) of the protein (atom color type) and the bound peptide (red):** (**a**) chicken calmodulin and smMLCK (code entry 2O5G), (**b**) human centrin 2 and the centrin binding region of XPC (code entry 2GGM), (**c**) human BCL-XL protein and BAK (code entry 1BXL), (**d**) human E3 ubiquitin- protein ligase MDM2 and p53 tumor transactivation domain (code entry 1YCR), (**e**) rabbit cardiac troponin C and cardiac troponin I (code entry 1A2X).

The interacting residues of the proteins and bound peptides, identified with ContPro [29], are shown in Figures 1 and 4 and Table 1. The results reveal that usually hydrophobic residues such as TRP, LEU, ILE, PHE, VAL, MET are involved in the interactions. The presence of hydrophobic residues suggests a favorable interaction with terphenyl-like molecules anchoring in the hydrophobic cavities. Most of the residues involved in the interactions between the proteins and alpha-helices are hydrophobic for both partners, as also observed in other studies [38]. We notice several key residues involved in the interaction of the same protein with different peptide partners. For example, in the case of calmodulin, PHE92, MET124, PHE141, MET144 and MET145 are involved in most of the peptides’ interactions. These residues can thus be considered as key for the interaction with terphenyl and its derivatives, or other alpha-helix mimetics. We noticed the presence of MET residues in most of the alpha-helix binding pockets analyzed here. In a recent study, MET residues have not been identified to be a part of hot spot amino acids, in particular in alpha-helix mediated protein interfaces [39]. However, our analysis clearly indicates their presence in positions that are key for the interaction with the alpha-helical partner. Furthermore, Ma and Nussinov [40] have also concluded that the amino acids TRP, MET, and PHE are important for protein-protein interactions. They showed that TRP/MET/PHE residues play roles in the dimerization of the transcriptase (p51/p66) and in cell-fusion processes, including the gp120-CD4 interaction and the gp41 six-helix bundle formation. They suggested that polarizability of MET allows it to assume roles of both hydrophobic and hydrophilic residues [40]. Further, its larger flexibility compared to other hydrophobic residues may facilitate the plasticity of hydrophobic binding pockets allowing to accommodate different ligands [27].

**Figure 5:**
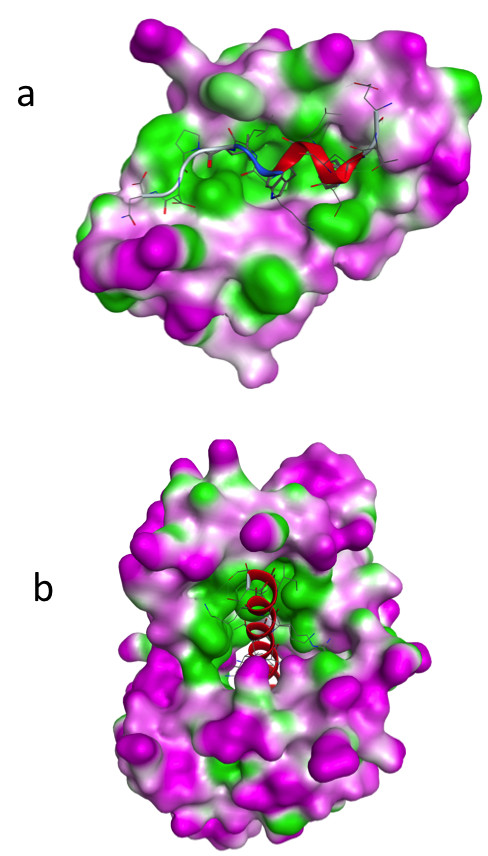
**Surface lipophilicity (shown in green) of alpha-helix binding proteins computed using MOE.** (**a**) Human E3 ubiquitin-protein ligase MDM2 (PDB code 1YCR), (**b**) Scherffelia dubia centrin (PDB code 3KF9).

We used Fpocket [33] and CASTp [32] to calculate geometrical and physicochemical characteristics of the binding pockets taking into account the protein residues interacting with the alpha-helical peptides. The overall hydrophobic character of the binding pockets is again clearly identified. Yet, some specificity is also observed, several pockets show high hydrophobicity score but low local hydrophobic density, or vice versa, demonstrating that the hydrophobic patches are not always regularly distributed in the binding pockets. For example, 1YCR and 3KF9 have similar hydrophobicity scores but high and low calculated hydrophobic density, respectively. The differences of the hydrophobicity distribution are illustrated in Figure 5.

The volumes of the detected pockets in the peptide-binding regions computed with CASTp are given in Table 3. The average volume of the sub-cavities present at the PPI interfaces found by Fuller et al [41] was ~60 **Å**^**3**^. Sonavane & Chakrabarti [42] found PPI pocket volumes to be up to ~330 **Å**^**3**^. We found similar volumes to those reported in Bourgeas et al. [43]. Taking into account the various algorithms and different concepts for binding pocket definition, such differences for the computed volumes can be expected. Several small cavities are present in the binding region (seen in Figure 2 and Figure 5), as it has been previously observed for other targeted PPI interfaces [39]. For the proteins studied here, the presence of several small hydrophobic cavities in the alpha-helix binding region seems to be a typical surface feature guiding the anchoring of hydrophobic residues from the peptide side. Such characteristics can also facilitate targeting PPI mediated by alpha-helices by small molecules containing hydrophobic anchors (as terphenyl or other mimetics).

**Table 3:** Geometrical and physicochemical characteristics of the identified pockets

**Protein code PDB**	**Volume (Å**^**3**^**)**	**Hydrophobicity score**	**Local hydrophobic density**
Chicken calmodulin	312.0	68.86	43.00
2O5G			
Human calmodulin	203.0	68.86	42.00
1ZUZ			
Human calmodulin	219.8	59.62	40.00
2VAY			
Human calmodulin	226.4	61.00	39.32
3DVE			
E.coli calmodulin	317.9	56.63	40.15
1QTX			
Rat calmodulin	310.6	56.62	43.78
2HQW			
Human centrin 2	147.9	41.47	32.00
2GGM			
Human centrin 2	210.9	39.93	35.08
2K2I			
Scherffelia dubia centrin	221.5	58.19	31.00
3KF9			
Human BCL-XL	321.5	36.91	42.04
1BXL			
Human E3 ubiquitin-protein ligase MDM2	201.9	51.18	55.20
1YCR			
Rabbit cardiac troponin C	213.1	63.07	39.15
1A2X			

**Figure 6:**
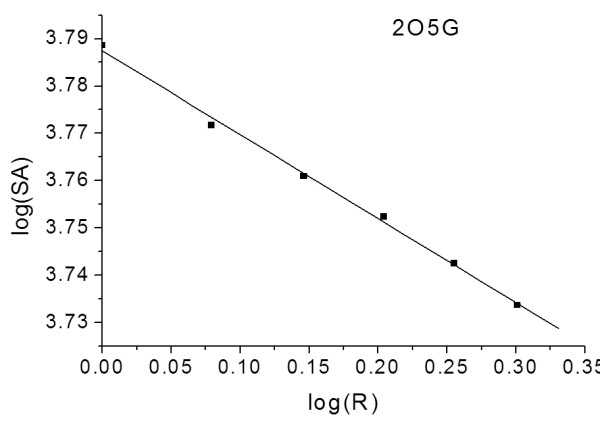
Double logarithmical plot of the surface area versus probe radii for chicken calmodulin (PDB code 2O5G).

Further, we decided to explore the roughness of the alpha-helix binding sites. The methodology implemented to calculate the fractal surface dimensions, used for the roughness evaluation, is illustrated in Figure 6 for the global surface roughness of chicken calmodulin. The fractal global surface dimension and the fractal local surface dimension for the binding site of chicken calmodulin are calculated to be D_S_=2.238; ± 0.006 and D_L_= 2.616 ± 0.072, respectively. The global and local fractal dimensions for the other proteins are given in Table 4. Our results and other previously published data [44-47] suggest that the global fractal dimension of protein surface is about 2. The local surface fractal dimensions for the binding cavities are computed to be larger than the global surface fractal dimensions for all studied proteins. This reflects the higher roughness of the binding site and its more complex shape and that can be considered as important for ligand binding. The most important differences between D_S_ and D_L_ are obtained for human calmodulin (2VAY), centrin (3KF9, 2K2I), BCL-XL (1BXL), MDM2 (1YCR) and troponin C (1A2X). It has been experimentally demonstrated that human calmodulin [18], BCL-XL [19,20] and MDM2 [21,22] interact with terphenyl or its derivatives. Recently, we suggested a possible binding of terphenyl 2, which mimics the relative positions of the side chains of residues TRP848, LEU851, LEU855 of the XPC peptide, into human centrin 2 following our energetic and conformational flexibility analysis performed for the alpha-helical peptide-binding pocket of centrin 2 [27]. The D_L_ value for the peptide-binding site of troponin C shows rougher surface than the entire protein, similarly to the above listed terphenyl-binding proteins.

**Table 4:** **Global (D**_**S**_**) and local (D**_**L**_**) surface fractal dimensions of investigated proteins**

**Code PDB**	**D**_**S**_	**D**_**L**_
2O5G	2.238 ± 0.006	2.616 ± 0.072
1ZUZ	2.181 ± 0.007	2.487 ± 0.058
2VAY	2.183 ± 0.006	2.757 ± 0.108
3DVE	2.217 ± 0.003	2.418 ± 0.040
1QTX	2.302 ± 0.002	2.494 ± 0.069
2HQW	2.172 ± 0.002	2.454 ± 0.082
2GGM	2.247 ± 0.004	2.373 ± 0.018
2K2I	2.167 ± 0.008	2.892 ± 0.124
3KF9	2.179 ± 0.006	2.892 ± 0.153
1BXL	2.230 ± 0.007	2.696 ± 0.225
1YCR	2.173 ± 0.014	2.708 ± 0.055
1A2X	2.177 ± 0.005	2.624 ± 0.032

**Figure 7:**
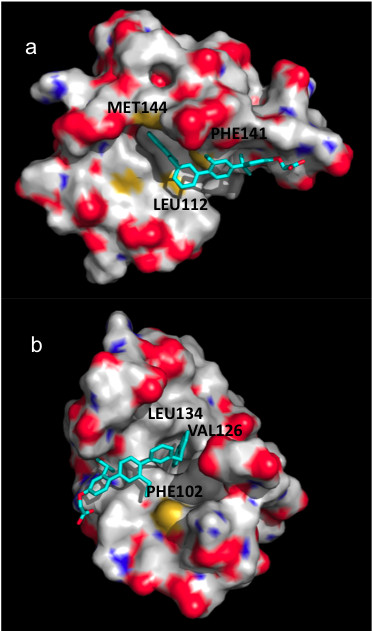
**Best scored docking poses of terphenyl.** The poses after docking-scoring with AutoDock are shown in cyan. (**a**) chicken calmodulin, code entry 2O5G, (**b**) rabbit cardiac troponin C, code entry 1A2X.

Taking into consideration the sequence and structural homology of troponin C and calmodulin and other physicochemical similarities of the binding sites as discussed above, we decided to probe putative terphenyl binding into troponin C. We performed docking of terphenyl 2 into the peptide-binding sites of calmodulin and troponin C using AutoDock. The best scored docking poses are shown in Figure 7. The terphenyl orientations in the best scored poses correspond to the position of the bound alpha-helical peptides shown in Figure 2. The predicted interaction energies of -7.98 and -8.18 kcal/mol for terphenyl binding in calmodulin and troponin C, respectively, suggest favorable interactions with the two proteins.

In the light of the results obtained here, it is now interesting to discuss the physicochemical properties of known PPI modulators, such as terphenyl. In a previous work [10] we gathered a set of 66 PPI inhibitors among which some terphenyl derivatives and other inhibitors of alpha-helix mediated PPI were present. In that work we demonstrated the more hydrophobic character of these compounds but also their bigger size. Interestingly, we also showed the importance of a critical number of aromatic bonds and some specific molecular shapes (T-shaped, star-shaped, or L-shaped compounds), among which some correspond to terphenyl derivatives. The present work therefore confirms that such genuine properties on the ligand side seem to be cavity-driven, and that these small molecules must possess certain properties in order to efficiently modulate an alpha-helix mediated PPI and to mimic the native partner and its properties.

## Conclusions

Modulating protein-protein interactions using small molecules based on surface recognition has been a field of increasing interest during the last decade. PPI interfaces are very complex and need to be analyzed in order to be efficiently targeted for drug discovery purposes. Designed compounds must bind with high affinity and selectivity to the target protein. The low sequence identity found between some of the analyzed proteins suggests that there are no sequence requirements for the ability of proteins to bind alpha-helical peptides and consequently small-molecule mimetics.

From the structural point of view, all investigated proteins show larger surface fractal dimensions for the peptide-binding pockets than the entire protein surface reflecting the higher complexity of the shape of the binding sites. Also, the presence of several hydrophobic patches at the protein surface seems to be an important property related to the ability of the protein to bind alpha-helical peptides and mimetics. Furthermore, we showed that hydrophobicity is not uniformly distributed across different alpha-helix binding pockets and that its distribution can be used to identify hydrophobic hot spots.

Many similarities between the binding sites studied here are observed and terphenyl or its derivatives binding to various alpha-helix binding proteins can be suggested. However, targeting various PPI complexes by similar small molecules can rise selectivity problems in the context of drug discovery or chemical biology projects. Thus, the specificities found here for different binding sites, e.g. key residues, roughness and local hydrophobic density, can be further exploited to optimize terphenyl-like ligands in order to improve their selectivity.

## Abbreviations

PPI: Protein-Protein interactions; smMLCK: smooth muscle myosin light chain kinase; CaM: Calmodulin; HsCen2: Human centrin 2; PDE: 3'-5'-cyclic nucleotide phosphodiesterase; XPC: Xeroderma pigmentosum group C.

## Competing interests

The authors declare that they have no competing interests.

## Authors’ contributions

AI carried out the sequence alignment and binding pockets analysis. DC carried out the fractal calculations. AI and DC drafted the manuscript. VM carried out the volume calculations and docking analysis. OS participated in the protein-protein interface analysis and discussion writing. MAM designed and coordinated the study. All authors participated in manuscript writing and approved the final manuscript.

## Pre-publication history

The pre-publication history for this paper can be accessed here:

http://www.biomedcentral.com/2050-6511/14/31/prepub
